# Homoharringtonine interacts synergistically with bortezomib in NHL cells through MCL-1 and NOXA-dependent mechanisms

**DOI:** 10.1186/s12885-018-5018-x

**Published:** 2018-11-16

**Authors:** Tri Nguyen, Rebecca Parker, Yu Zhang, Elisa Hawkins, Maciej Kmieciak, William Craun, Steven Grant

**Affiliations:** 1 Division of Hematology/Oncology, Virginia Commonwealth University Richmond, Room 229 Goodwin Research Building, 401 College Street, Richmond, VA 23229 USA; 2 Palliative Care, Virginia Commonwealth University Richmond, Richmond, VA USA; 3 Department of Biochemistry, Virginia Commonwealth University Richmond, Richmond, VA USA; 4 Human and Molecular Genetics, Virginia Commonwealth University Richmond, Richmond, VA USA; 5 Massey Cancer Center, Virginia Commonwealth University Richmond, Richmond, VA USA

**Keywords:** Homoharringtonine (Omacataxine), Bortezomib, Mantle cell lymphoma, Diffuse large B-cell lymphoma

## Abstract

**Background:**

Interactions between the protein synthesis inhibitor homoharringtonine (HHT) and the proteasome inhibitor bortezomib were investigated in DLBCL and mantle cell lymphoma cells (MCL).

**Methods:**

Various DLBCL and MCL cells were exposed to HHT and bortezomib alone or together after which apoptosis and signaling pathway perturbations were monitored by flow cytometry and Western blot analysis. Xenograft mouse models were used to assess tumor growth and animal survival.

**Results:**

HHT and bortezomib co-administration synergistically induced apoptosis in GC-, ABC- and double-hit DLBCL cells. Similar interactions were observed in MCL cells and in primary lymphoma cells. HHT/bortezomib co-administration diminished binding of MCL-1 to both BAK and NOXA. Knock-down of NOXA significantly diminished lethality whereas MCL-1 knock-down or ectopic NOXA expression increased cell death. Notably, HHT/bortezomib lethality was dramatically reduced in BAK knockout or knockdown cells. Finally, HHT/bortezomib co-administration significantly improved survival compared to single agents in GC- and ABC- xenograft models while exhibiting little toxicity.

**Conclusions:**

These findings indicate that HHT and bortezomib cooperate to kill DLBCL and MCL cells through a process involving MCL-1 down-regulation, NOXA up-regulation, and BAK activation. They also suggest that a strategy combining HHT with bortezomib warrants attention in DLBCL and MCL.

**Electronic supplementary material:**

The online version of this article (10.1186/s12885-018-5018-x) contains supplementary material, which is available to authorized users.

## Background

Diffuse large B-cell lymphoma (DLBCL) is a form of non-Hodgkin’s lymphoma (NHL) that afflicts approximately 23.000 patients/year in the US [1]. Despite recent advances such as the introduction of effective new targeted therapies (e.g., ibrutinib) [2] and an improved understanding of the molecular pathogenesis of this disorder [3], patients with relapsed/refractory disease have a dismal prognosis. In addition, outcomes in certain genetic sub-types e.g., ABC (activated B-cell) versus GC (germinal center) DLBCL are inferior [4, 5], and patients with double- (or triple-) hit lymphomas displaying increased expression of BCL-2, BCL-6, and/or c-Myc do particularly poorly [6]. Mantle cell lymphoma (MCL) is an aggressive form of lymphoma which also carries a relatively poor prognosis [7]. Consequently, newer and more effective treatment strategies are urgently needed for these diseases.

Bortezomib is an inhibitor of the 20S proteasome, and by extension, the ubiquitin-proteasome system (UPS), which is responsible for degradation of diverse cellular proteins and maintenance of protein homeostasis [8]. It is approved for use in multiple myeloma as well as in MCL, in which single-agent activity is 30% [9]. Addition of bortezomib to standard chemotherapy may also be of benefit in certain DLBCL sub-types e.g., ABC-DLBCL [10]. The mechanism of resistance of neoplastic cells e.g., myeloma to bortezomib is not known with certainty, but accumulation of anti-apoptotic proteins e.g., MCL-1 due to interference with degradation has been implicated [11].

Homoharringtonine (HHT or omacetaxine mepesuuccinate, Synribo®) is an inhibitor of translation elongation and protein synthesis [12, 13]. It is a semisynthetic derivative omacetaxine mepesuccinate which has been approved for the treatment of patients with chronic myelogenous leukemia (CML) resistant to tyrosine kinase inhibitors [14, 15]. Its ability to disrupt protein synthesis leads to down-regulation of short-lived proteins, including MCL-1 [16]. Indeed, the lethal effects of HHT in various malignant hematopoietic cells e.g., leukemia has been related to diminished expression of this protein [12, 17, 18].

The potential role of MCL-1 in conferring bortezomib resistance [11, 19] supports the use of HHT in conjunction with this agent. In fact, studies in multiple myeloma cells indicate that HHT potentiates bortezomib activity through multiple mechanisms, including MCL-1 down-regulation and interference with stromal cell factors, among others [20]. Currently, no information exists regarding whether HHT might enhance bortezomib activity in NHL cells, and the mechanisms that may underlie such a phenomenon. Here we report that HHT synergistically enhances the activity of bortezomib against diverse lymphoma cell types (including primary and double-hit DLBCL cells) both in vitro and in vivo through mechanisms involving MCL-1 down-regulation, NOXA up-regulation, and activation of BAK. Together, these findings raise the possibility of combining HHT and bortezomib in the setting of NHL.

## Methods

### Cells

All cell lines were kindly provided or purchased and cultured as described previously [21].

### Immunoblot and immunoprecipitation

Western blot analysis was carried out as previously described [21, 22]. Primary antibodies used in these studies were: cleaved PARP, cleaved caspase-3, BCL-_XL_, BIM (Cell Signaling Technology, Danvers, MA), MCL-1 (BD Biosciences, San Jose, CA), α-tubulin (EMD Millipore, Billerica, MA), BAX (N20), BAK (G23), actin (Sigma-Aldrich, St. Louis, MO), NOXA (Enzo Life Sciences, Farmingdale, NY).

### Plasmids and transfection

Knockdown MCL-1 and NOXA plasmids were purchased from Dharmacon (Open Biosystem). NOXA/Flag plasmid was kindly provided by Dr. Harada [23]. Luciferase or scrambled shRNA/pLKO.1 was used as control. Lentivirus production was generated using Lipofectamine 3000 (Invitrogen, ThermoFisher Scientific, NJ) following the manufacturer’s protocol.

### Reagents

Homoharringtonine (Omacetaxine®) was provided by Teva Pharmaceutical Industries Ltd. Bortezomib was purchased from Chemietek (Indianapolis)*.* BOC-D-fmk was purchased from Abcam. All agents were formulated in DMSO and stocked in − 80 °C for in vitro use.

### Quantitative real-time PCR

Quantitative real-time PCR (qPCR) analysis using TaqMan gene expression assays and a 7900HT real-time PCR system (Applied Biosystems, Foster City, CA) was performed to quantify mRNA levels of human MCL-1. Briefly, total RNA was isolated by using TRIzol reagent (Invitrogen, Carlsbad, CA) according to the manufacturer’s instructions. Genomic DNA was digested with DNase I (amplification grade; Invitrogen). cDNA was synthesized from 1 μg of total RNA by using a High Capacity cDNA reverse transcription kit (Applied Biosystems). One microliters of cDNA was employed for qPCR assays (TaqMan gene expression assays). Assay identification numbers for MCL-1 were Hs03043899_m1. References for quantitation were human β-actin and glyceraldehyde-3-phosphate dehydrogenase (GAPDH) (Applied Biosystems). Data were analyzed by using SDS 2.3 software.

### In vivo studies

NOD/SCID-γ mice were subcutaneously injected in the flank with 10 × 10^6^ luciferase-expressing U2932 or SU-DHL4 cells. Tumor volume was followed and measured with calipers using the following formula: tumor volume (mm^3^) = length (mm) × width (mm)^2^/2. Omacetaxine (1 mg/kg, 5 days a weeks) and bortezomib (0.75 mg/kg, twice a week) was administered via intraperitoneal (i.p.). Control animals were injected with equal volumes of vehicle.

Mice were monitored for tumour growth with caliper and the imaging system by IVIS 200 (Xenogen Corporation, Alameda, CA).

### Cell growth and viability, assessment of apoptosis and flow cytometry, collection and processing of primary normal CD34^+^, lymphoma patient cells and statistical analysis

All procedures and experiments were followed and performed as previously described in detail [21, 22, 24].

## Results

**Fig. 1:**
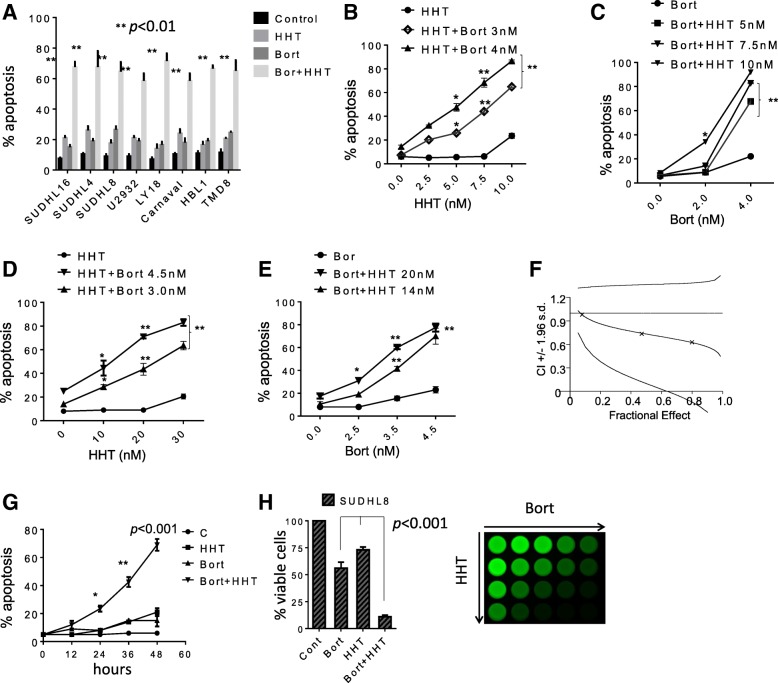
HHT dramatically increases bortezomib lethality and inhibits cell growth in DLBCL cells*.* **a** Various NHL cell lines SU-DHL16, SU-DHL4, SU-DHL8 (GC subtype), HBL-1, U2932 (ABC-subtype), OCI-LY18, Carnaval (double-hit) were exposed to HHT (10, 15, 12, 15, 30, 10, 10 nmol/L respectively) and bortezomib (1.5, 4, 2.5, 2, 4.5, 3, 2.5 nmol/L respectively) alone or together for 48 h, after which cell death was assessed by 7-AAD. *p < 0.05, **p < 0.01, significantly greater than values for single agent treatment. For these and subsequent studies, values represent the means ± S.D. for experiments performed in triplicate on at least 3 separate occasions. **b** SU-DHL16 cells were exposed to the indicated concentration of HHT in the presence or absence of bortezomib for 48 h, after which cell death was assessed by 7-AAD. **c** SU-DHL16 cells were exposed to the indicated concentration of bortezomib in the presence or absence of HHT for 48 h, after which cell death was assessed by 7-AAD. **d** SU-DHL8 cells were exposed to the indicated concentration of HHT in the presence or absence of bortezomib for 48 h, after which cell death was assessed by 7-AAD. **e** SU-DHL8 cells were exposed to the indicated concentration of bortezomib in the presence or absence of HHT for 48 h, after which cell death was assessed by 7-AAD. *p < 0.05, **p < 0.01, significantly greater than values for single agent treatment. **f** SU-DHL-8 cells were treated with a range of HHT and bortezomib concentrations administered at a fixed ratio. At the end of 48 h, the percentage of cell death was determined by monitoring 7AAD^+^ cells. CI values were determined in relation to the fractional effect by using Calcusyn software. CI values less than 1.0 correspond to synergistic interactions. **g** SU-DHL8 cells were treated with HHT (12 nmol/L) or bortezomib (3.5 nmol/L) individually or in combination for the indicated intervals, after which the extent of cell death was determined by 7-AAD uptake and flow cytometry. **h** Cells were exposed to HHT and bortezomib as described above alone or together for 48 h, after which cells were enumerated by hemocytometer (left panel, *p* < 0.001, significantly greater than values for single agent treatment). SU-DHL-8 were exposed to increasing concentrations of HHT and bortezomib, after which cell growth and viability were evaluated using the CellTiter-Glo Luminescent assay (right panel)

Co-administration (48 h) of HHT (5–40 nM) with bortezomib (1–5 nM) in diverse NHL lines e.g., SU-DHL-16, SU-DHL-4, SU-DHL-8 (GC), U2932, TMD8, HBL-1 (ABC), including double-hit (OCI-LY18, Carnaval) resulted in a pronounced increase in apoptosis (Fig. 1a). Dose-response studies in SU-DHL16 (GC) cells revealed significant increases in cell death at HHT and bortezomib concentrations as low as 7.5 nM or 4 nM respectively (Fig. 1b-c). Similarly, SU-DHL8 cells showed significant increases in cell death at HHT and bortezomib concentrations as low as 20 nM or 3.5 nM respectively (Fig. 1d-e). Median Dose Effect analysis yielded CI values < 1.0, denoting synergistic interactions (Fig. 1f). Time course studies showed that significant increases in cell death were observed at 24 h of co-incubation, and increased further over the ensuing 24 h (Fig. 1g). Similar results were observed in SU-DHL4 (Additional file 1A) and double-hit OCI-LY18 DLBCL cells; Additional file 1B-E). Finally, equivalent results were obtained when viable cell number and MTT assays were monitored (Fig. 1f).

**Fig. 2:**
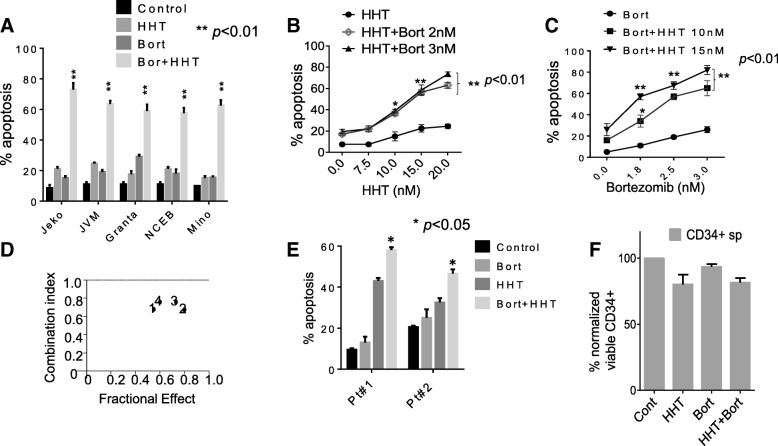
Co-treatment with HHT and bortezomib synergistically induces cell death in mantle cell lymphoma, and primary patient specimens, but not normal CD34^+^ bone marrow cells. **a** Granta-519, Jeko-1, JVM, NCEB, Mino cells were exposed to HHT (10, 20, 15, 15, 15 nmol/L respectively) and bortezomib (2.5, 3.5, 2.5, 2.5, 3 nmol/L respectively) alone or in combination for 48 h, after which cell death was assessed by 7-AAD. **p < 0.01, significantly greater than values for single agent treatment. **b** Jeko cells were exposed to the indicated concentration of HHT in the presence or absence of bortezomib for 48 h after which cell death was assessed by 7-AAD. **p < 0.01, significantly greater than values for single agent treatment. **c** Jeko-1 cells were treated with a range of HHT and bortezomib concentrations. At the end of this period, the percentage of 7AAD^+^ cells was determined by flow cytometry. CI values less than 1.0 reflect synergistic interactions. **d** Jeko-1 cells were treated with a range of HHT and bortezomib concentrations administered at a fixed ratio. The percentage of cell death was determined by monitoring 7AAD^+^ cells at 48 h. CI values were determined in relation to the fractional effect by using Calcusyn software. CI values less than 1.0 correspond to synergistic interactions. **e** Mononuclear peripheral blood cells from a primary double-hit DLBCL (pt#1) and a NHL follicular (pt#2) lymphoma were exposed to HHT (15–20 nmol/L) or bortezomib (4 nmol/L) individually in combination for 48 h, after which the percentage of apoptotic cells was determined by annexin V/PI (*p < 0.05, significantly greater than values for single-agent treatment). **f** Mononuclear cord blood cells were isolated and exposed to HHT (20 nmol/L) or bortezomib 5 nmol/L individually or in combination for 48 h, after which viable (non-apoptotic) CD34^+^ cells was determined by annexin V/PI positivity. *P* values for the combination were > 0.05, not significantly different compared to values for either agent alone

Parallel studies were performed in mantle cell lymphoma cells, where co-administration (48 h) of HHT and bortezomib resulted in marked increase in apoptosis in 5 MCL lines (Fig. 2a). Dose responses in Jeko1 cells were similar to those obtained in DLBCL cells i.e., significant increases in cell death were observed with HHT concentrations ≥10 nM and bortezomib concentrations ≥1.8 nM (Fig. 2b-c). Similar results were observed in NCEB cells (Additional file 1-F). Median Dose Effect analysis confirmed synergistic interactions in Jeko (Fig. 1d). Finally, studies performed in primary cells obtained from peripheral blood cells of 2 patients (#1 double-hit DLBCL; #2 follicular lymphoma) revealed significant increases in cell death with concomitant HHT/bortezomib exposure compared to single-agent treatment (24 h; Fig. 2e). Similar exposures minimally induced cell death in normal CD34^+^ cells (Fig. 2f).

**Fig. 3:**
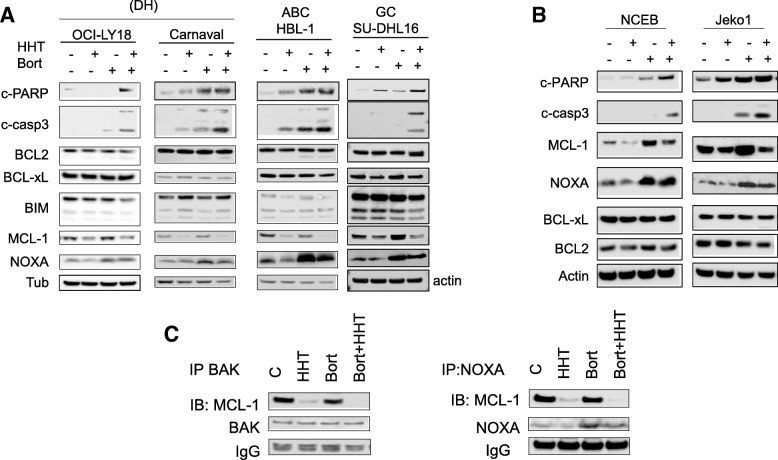
The homoharringtonine/bortezomib regimen induces caspase activation and alters the NOXA/MCL1 ratio. **a**, **b** OCI-LY18, Carnaval, HBL1 and NCEB cells were treated with HHT (10 to 20 nM) alone or with bortezomib (2 to 3 nmol/L) for 24 h after which cells were lysed and proteins extracted. Expression of the indicated proteins was determined by Western blotting using the indicated antibodies. Each lane was loaded with 25 μg of protein; blots were stripped and re-probed with tubulin/actin to ensure equivalent loading and transfer. Results are representative of three replicate experiments. **c** SU-DHL-4 cells were exposed the HHT (25 nmol/L) and bortezomib (4 nmol/L) individually or together for 24 h after which cells were lysed and subjected to immunoprecipitation using BAK or NOXA Abs. The immunoprecipitates were separated by SDS-PAGE and immunoblotted with MCL-1 (left and right panel)

Western blot analysis was employed to monitor expression of BCL-2 family members in response to the HHT/bortezomib regimen. Combined treatment (20 h) of GC-DLBCL (SU-DHL16), double-hit DLBCL cells (OCI-LY18, Carnaval) or ABC-DLBCL (HBL-1) resulted in increased caspase-3 cleavage but little change in the expression of BCL2, BCL-xL, or BIM (Fig. 3a). Notably, HHT in combination with bortezomib resulted in a further reduction in levels of MCL-1. In addition, bortezomib alone or with HHT sharply increased expression of the pro-apoptotic protein NOXA. Similar results were observed in the case of Jeko-1 and NCEB mantle cell lymphoma cells in which bortezomib alone clearly up-regulated MCL-1 expression, and this effect was attenuated by HHT (Fig. 3b). To determine whether any of these perturbations were secondary to caspase-mediated degradation, OCI-LY18 and Carnaval cells were incubated with HHT + Bort in the presence or absence of the broad-spectrum caspase inhibitor BOC-D-fmk 5 μmol/L. Addition of BOC-D-fmk did not change HHT/Bort-mediated down-regulation of MCL-1 (Additional file 2). These findings suggest that HHT/Bort-induced changes in signaling proteins in all likelihood do not represent a consequence of cell death. Finally, immunoprecipitation studies in SU-DHL-4 cells revealed that co-administration of HHT and bortezomib diminished binding of MCL-1 to BAK and NOXA (Fig. 3c).

Studies were then undertaken to characterize the basis by which HHT down-regulated MCL-1 expression. As shown in Additional file 3A, HHT alone reduced MCL-1 expression by 8 h in both SU-DHL4 and 16 cells. However, RT-PCR analysis revealed that HHT induced, if anything, an increase in MCL-1 mRNA (Additional file 3B). In addition, co-administration of the transcriptional inhibitor actinomycin resulted in a further decline in MCL-1 levels (Additional file 3C), suggesting an alternative mechanism of action. In contrast, the translational inhibitor cyclohexamide had little effect on HHT-mediated MCL-1 down-regulation (Additional file 3D), consistent with a common mechanism of action. Together, these findings argue that HHT acts to down-regulate MCL-1 in these cells through a post-transcriptional mechanism.

**Fig. 4:**
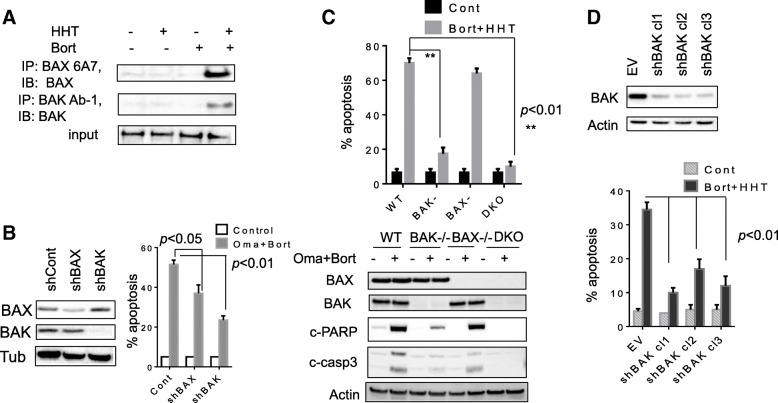
BAX and particularly BAK play significant role in the lethality of the HHT/bortezomib regimen. **a** OCI-LY18 cells were exposed to HHT (12 nM) and bortezomib (3 nM) alone or in combination for 24 h after which cells were lysed in buffer containing 1% CHAPS. Conformational changed BAX and BAK proteins were immunoprecipitated using anti-BAX-6A7 and anti-BAK-Ab1 Abs respectively, and subjected to Western blot analysis using polyclonal BAX or BAK Abs. **b** U2932 cells were transfected with shRNA constructs designed against BAX and BAK. These U2932/shBAX, shBAK and shControl cells were exposed to HHT (25 nM) and bortezomib (4 nM) for 48 h after which cells were analyzed flow cytometry. **c** MEF, MEF BAK−/−, MEF BAX−/− and MEF DKO were exposed to HHT (40 nmol/L) and bortezomib (5 nmol/L) after which cell death was assessed by 7-AAD (upper panel) or the cells were subject to western blot (lower panel). **d** SU-DHL-4 cells were transfected with BAK/shRNA constructs. Three SU-DHL-4/shBAK clones were selected. These and shControl cells were exposed to HHT (25 nM) and bortezomib (4 nM) for 48 h after which cells were analyzed flow cytometry

The role of the pro-apoptotic multi-domain proteins BAX and BAK on responses to the HHT/bortezomib regimen were then examined. While exposure of OCI-LY18 cells to HHT or bortezomib individually had little effect on BAX or BAK conformational change, combined treatment robustly increased activation of both (Fig. 4a). Furthermore, shRNA knock-down of BAX in U2932 cells modestly but significantly diminished HHT/bortezomib lethality whereas BAK knock-down sharply reduced cell killing (Fig. 4b). Parallel studies performed in BAK, BAX and double-knock-out (DKO) MEF cells revealed that BAK KO or DKO dramatically reduced lethality whereas BAX KO had little effect (Fig. 4c, upper panel). Consistent results were obtained when PARP and caspase-3 cleavage were monitored (Fig. 4c, lower panel). Finally, shRNA knock-down of BAK in SU-DHL-4 cells (Fig. 4d, upper panel) significantly diminished HHT/bortezomib lethality (*p* < 0.01; Fig. 4d, lower panel). Together, these findings argue that BAK activation plays a significant functional role in HHT/bortezomib lethality.

**Fig. 5:**
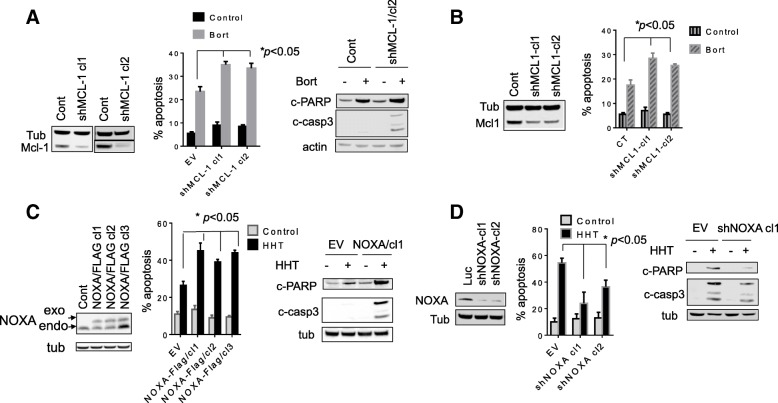
Genetic inhibition of MCL-1 or overexpression of NOXA renders cells significantly more sensitive to bortezomib*.* **a** SU-DHL-4 cells were transfected with shRNA constructs designed against MCL-1. Two clones of shRNA MCL-1 were selected. These 2 clones of SU-DHL-4/shMCL1 and shControl cells were exposed to bortezomib (4 nM) for 48 h after which cells were analyzed by flow cytometry and western blot. **b** Similarly, SU-DHL-16 cells were transfected with shRNA constructs designed against MCL-1. SU-DHL-16/shMCL1 and shControl cells were exposed to bortezomib (1.5 nM) for 48 h after which cells were analyzed flow cytometry and Western blot. **c** SU-DHL-16 cells were transfected with NOXA/Flag constructs. Three clones with overexpression of NOXA were selected. These clones were exposed to HHT (10 nM) for 48 h then analyzed by flow cytometry. **d** SU-DHL-16 cells were transfected with shNOXA constructs. Two clones with knockdown of NOXA were selected. These clones were exposed to HHT (10 nM) for 48 h then analyzed by flow cytometry

To evaluate the functional significance of MCL-1 down-regulation in HHT/bortezomib lethality, SU-DHL-4 MCL-1 shRNA knock-down clones were generated (shMCL-1 cl1 and cl2; Fig. 5a, left panel). These clones were significantly more sensitive than empty-vector controls to bortezomib-induced apoptosis (Fig. 5a, middle panel) and PARP/caspase-3 cleavage (Fig. 5a, right panel). Similar results were obtained in SU-DHL-16 cells in which MCL-1 was knocked down (Fig. 5b). These findings argue that MCL-1 down-regulation by HHT is likely to increase bortezomib lethality.

To assess the impact of NOXA up-regulation in HHT/bortezomib activity, SU-DHL-16 cells were engineered to over-express NOXA (Fig. 5c, left panel). Notably, each of the three over-expressing clones was significantly more sensitive to HHT than empty-vector controls (Fig. 5c, middle and right panels). Conversely, NOXA shRNA knock-down cells were significantly less sensitive to HHT-induced apoptosis than their empty-vector counterparts (Fig. 5d).

**Fig. 6:**
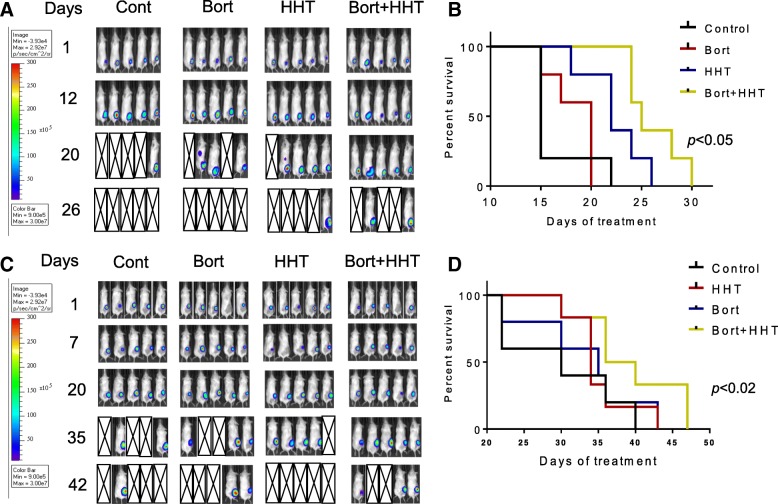
Co-treatment with HHT and bortezomib suppresses tumor growth in murine xenograft models and prolongs animal survival. NOD/SCID-γ mice were subcutaneously inoculated in the right rear flank with 10 × 10^6^ SU-DHL-4/Luc (**a**) and U2932/Luc (**b**) cells which stably express luciferase. Treatment was initiated after the tumor were visualized, measured, and randomly grouped 10 days after injection of tumor cells. HHT was administrated at a dose of 1 mg/kg by i.p 5 days a week. Bortezomib was administered at a dose of 0.75 mg/kg i.p twice a week. Control animals were administered equal volumes of vehicle. **a** Tumor growth (SU-DH-L4) was monitored twice weekly by injection of luciferin and imaged by the IVIS 200 imaging system. d = day, empty boxes represent deceased mice. **b** Kaplan–Meier analysis was performed to analyze survival of animals. The survival of mice treated with the combination was significantly prolonged compared to mice treated with single agents (*p* < 0.05). **c** Tumor growth (U2932) was monitored twice weekly by injection of luciferin and imaged by the IVIS 200 imaging system. d = day, empty boxes represent deceased mice. **b** Kaplan–Meier analysis was performed to analyze survival of animals. The survival of mice treated with the combination was significantly prolonged compared to mice treated with single agents (*p* < 0.02)

Finally, the in vivo activity of the HHT/bortezomib regimen was evaluated in two xenograft models. Co-administration of HHT (1 mg/kg 5d/wk) and bortezomib (0.75 mg/kg 2×/wk) reduced tumor growth and significantly increased survival in mice inoculated in the flank with SU-DHL-4 cells (*p* < 0.05) compared to single-agent treatment (Fig. 6a-b). Similar results were obtained in mice inoculated with double-hit U2932 cells (survival significantly greater than with single agents; *p* < 0.02; Fig. 6c-d). In neither model did the regimen induce significant weight loss (e.g., > 10%; Additional file 4) or other signs of toxicity.

## Discussion

The results of this study indicate that the translational inhibitor HHT interacts synergistically with bortezomib to induce apoptosis in vitro in diverse DLBCL and MCL types, and that the regimen is also effective in the in vivo setting. The mechanisms by which these agents interact are likely to be multi-factorial, and appear to involve down-regulation of MCL-1, up-regulation of NOXA, and activation of BAK. The bulk of pre-clinical data related to HHT involves CML models [13, 14], a disease for which this agent is approved in patients with TKI-resistant disease [15]. Recently, studies have suggested that HHT may enhance the lethality of bortezomib in multiple myeloma cells through mechanisms involving inactivation of AKT and NF-kB [20]. Reports of HHT in NHL models are very limited, although one study revealed that HHT lowered the threshold for apoptosis in a sub-set of DLBLC cells exposed to the BH3-mimetic and Bcl-2 antagonist venetoclax [25]. To the best of our knowledge, this represents the first description of HHT/bortezomib synergism in DLBCL and MCL models, diseases in which bortezomib may also play a useful role [9, 26].

It is likely that down-regulation of MCL-1 contributes to the observed interactions between bortezomib and HHT. In contrast to BCL-2, MCL-1 is a relatively short-lived protein, and interventions that block its synthesis e.g., transcriptional antagonists that inhibit CDK9/pTEFb trigger its down-regulation [27–29]. Analogously, inhibitors of translation have also been shown to diminish MCL-1 abundance [12, 30]. In this regard, HHT has been shown to down-regulate MCL-1 in human acute and chronic myeloid leukemia cells [13, 31], chronic lymphocytic leukemia cells [12], and MM cells [20]. The present results indicate that similar events occur in DLBCL and MCL cells. Notably, in addition to its effects on the proteasome and disruption of protein homeostasis, proteasome inhibitors such as bortezomib can induce cell death by preventing the down-regulation of pro-apoptotic proteins e.g., p53 [32, 33]. However, they may also spare certain anti-apoptotic proteins e.g., MCL-1, potentially leading to drug resistance [11]. The observation that shRNA knock-down of MCL-1 significantly increased bortezomib lethality argues that HHT-mediated MCL-1 down-regulation contributed functionally to the activity of this regimen.

The present results argue that NOXA up-regulation by bortezomib also plays a significant functional role in NHL cell death triggered by the HHT/bortezomib regimen. The ability of bortezomib to induce NOXA, contributing to cell death, has been described in several hematopoietic cell types, including CLL [34], MCL [35], and multiple myeloma cells [36]. Of note, NOXA has been implicated in destabilization of MCL-1 [37], raising the possibility of involvement of an amplification loop in HHT/bortezomib interactions. Additionally, co-administration of HHT markedly diminished the amount of MCL-1 co-immunoprecipitating with NOXA, potentially promoting NOXA pro-apoptotic actions [38, 39]. Whether this phenomenon reflects MCL-1 down-regulation or other as yet to be determined actions of HHT remains to be determined. In any case, the finding that enforced NOXA expression significantly increased and shRNA NOXA knock-down significantly reduced HHT lethality in DLBCL cells strongly implicates NOXA up-regulation in HHT/bortezomib synergism.

While co-administration of HHT and bortezomib induced conformational change/activation of the multi-domain pro-apoptotic proteins BAX and BAK, several lines of evidence argue that BAK activation was the primary basis for HHT/bortezomib lethality. In this context, both BAK and NOXA have been identified as critical determinants of bortezomib lethality in mesothelioma cells [40]. However, HHT/bortezomib lethality was minimally affected in BAX MEF KO cells, whereas it was essentially abrogated in their BAK KO counterparts. Moreover, BAX knock-down in DLBCL cells only modestly diminished HHT/bortezomib lethality, whereas BAK knock-down had a significantly greater effect. Of note, BAK is tethered and inactivated by MCL-1 and BAK can be activated by NOXA [41], raising the possibility that MCL-1 down-regulation and NOXA up-regulation cooperate to activate BAK and subsequently mitochondrial apoptosis.

The finding that ABC- and GC-type DLBCL cells were equally sensitive to the HHT/bortezomib regimen could reflect multiple factors, including the lack of NF-κB-dependent mechanisms underlying interactions between these agents (e.g., MCL-1 down-regulation). The regimen was also effective against double-hit DLBCL models characterized by c-Myc and Bcl-2 overepression, and associated with markedly inferior outcomes in the clinic [4]. Finally, the regimen was active against MCL, a disease for which curative therapy is generally lacking and in which single-agent bortezomib has been approved [9]. Significantly, each of these cell types was susceptible to regimens employing very low HHT and bortezomib concentrations (e.g., low nM), easily achieved in the plasma of patients receiving these agents [42]. Finally, the observation that the HHT/bortezomib regimen was well tolerated in two DLBCL xenograft models while significantly prolonging survival compared to single-agent administration is noteworthy.

## Conclusions

These findings indicate that HHT and bortezomib synergistically kill DLBCL and MCL cells through a process involving MCL-1 down-regulation, NOXA up-regulation, and BAK activation. The HHT/bortezomib regimen also significantly prolonged survival in DLBCL xenograft models compared to single-agent administeration. These findings argue that such a regimen warrants consideration in patients with high-risk, aggressive forms of DLBCL for whom satisfactory therapeutic options are lacking. Efforts to explore this possibility are underway.

## Additional files

Additional file 1: *HHT dramatically increases bortezomib lethality and inhibits cell growth in DLBCL cells.* A) SU-DHL4 cells were exposed to the indicated concentration of HHT in the presence or absence of 4 nM bortezomib for 48 h, after which cell death was assessed by 7-AAD. B) OCI-LY18 cells were exposed to the indicated concentration of HHT in the presence or absence of bortezomib for 48 h, after which cell death was assessed by 7-AAD. C) OCI-LY18 cells were exposed to the indicated concentration of bortezomib in the presence or absence of HHT for 48 h, after which cell death was assessed by 7-AAD. D) OCI-LY18 cells were treated with HHT (12 nmol/L) or bortezomib (3 nmol/L) individually or in combination for the indicated intervals, after which the extent of cell death was determined by 7-AAD uptake and flow cytometry. E) OCI-LY18 cells were treated with a range of HHT and bortezomib concentrations administered at a fixed ratio. At the end of 48 h, the percentage of cell death was determined by monitoring 7AAD^+^ cells. CI values were determined in relation to the fractional effect by using Calcusyn software. CI values less than 1.0 correspond to synergistic interactions. F) NCEB cells were exposed to the indicated concentration of HHT in the presence or absence of bortezomib for 48 h, after which cell death was assessed by 7-AAD. (PPTX 172 kb)

Additional file 2: *The caspase inhibitor BOC-D-fmk does not change HHT/Bort–mediated down-regulation of MCL-1*. OCI-LY18 and Carnaval cells were treated with HHT + Bort for 24 h either in the absence or presence of 5 μmol/L BOC-D-fmk. At the end of this period, cells were lysed and subjected to Western blot analysis using the indicated primary antibodies. Each lane was loaded with 25 μg of protein. Blots were stripped and reprobed with antitubulin antibodies to ensure equal loading and transfer of protein. Representative of two separate experiments. (PPTX 100 kb)

Additional file 3: *HHT inhibits MCL-1 expression through a post-transcriptional mechanism.* A. SU-DHL4 and SU-DHL16 cells were treated with HHT for 8 h after which cells were lysed and proteins extracted. Expression of the indicated proteins was determined by Western blotting using the indicated antibodies. B. SU-DHL4 and SU-DHL16 cells were treated with HHT for 8 h after which cells were extracted for mRNA. Relative levels of MCL-1 mRNA/GAPDH were calculated. C. SU-DHL4 and SU-DHL16 cells were pre-treated with actinomycin (2.5 μg/ml) for 30 min and then exposed to HHT 2 h (SU-DHL4 60 nM, SU-DHL16 20 nM) after which cells were lysed and proteins extracted. Expression of the indicated proteins was determined by western blott using the indicated antibodies. D. SU-DHL4 and SU-DHL16 cells were pre-treated with cyclohexamide (5 μg/ml) for 30 min and then exposed to HHT 2 h and 4 h (SU-DHL4 60 nM, SU-DHL16 20 nM) after which cells were lysed and proteins extracted. Expression of the indicated proteins was determined by western blot. (PPTX 236 kb)

Additional file 4: *Co-treatment with HHT and bortezomib does not cause significant weight loss in NSG mice.* A. Weights of each mouse in the flank model study (SU-DHL-4) were monitored twice a week, and the mean weights for each group were plotted against days of treatment (*p* > 0.05 = no significant difference for combination group values compared to single-agent treatment or controls). B. Weights of each mouse in the systemic model study (U2932) were monitored twice a week and the mean weights for each group were plotted against days of treatment (*p* > 0.05 = no significant differences were noted for the combination group values compared to single-agent treatment or the control group. (PPTX 134 kb)

## Abbreviations

DLBCL: Diffuse large B cell lymphoma
HHT: Homoharringtonine
MCL: Mantle cell lymphoma
NHL: Non-Hodgkin lymphoma
