# Supplementary material for: Homoharringtonine interacts synergistically with bortezomib in NHL cells through MCL-1 and NOXA-dependent mechanisms
Source: BMC Cancer. 2018 Nov 16;18:1129. doi: 10.1186/s12885-018-5018-x (PMC6240231; doi:10.1186/s12885-018-5018-x)
Supplement: Supplementary file 1 — HHT dramatically increases bortezomib lethality and inhibits cell growth in DLBCL cells. A) SU-DHL4 cells were exposed to the indicated concentration of HHT in the presence or absence of 4 nM bortezomib for 48 h, after which cell death was assessed by 7-AAD. B) OCI-LY18 cells were exposed to the indicated concentration of HHT in the presence or absence of bortezomib for 48 h, after which cell death was assessed by 7-AAD. C) OCI-LY18 cells were exposed to the indicated concentration of bortezomib in the presence or absence of HHT for 48 h, after which cell death was assessed by 7-AAD. D) OCI-LY18 cells were treated with HHT (12 nmol/L) or bortezomib (3 nmol/L) individually or in combination for the indicated intervals, after which the extent of cell death was determined by 7-AAD uptake and flow cytometry. E) OCI-LY18 cells were treated with a range of HHT and bortezomib concentrations administered at a fixed ratio. At the end of 48 h, the percentage of cell death was determined by monitoring 7AAD+ cells. CI values were determined in relation to the fractional effect by using Calcusyn software. CI values less than 1.0 correspond to synergistic interactions. F) NCEB cells were exposed to the indicated concentration of HHT in the presence or absence of bortezomib for 48 h, after which cell death was assessed by 7-AAD. (PPTX 172 kb) [file 12885_2018_5018_MOESM1_ESM.pptx]

## Slide 1
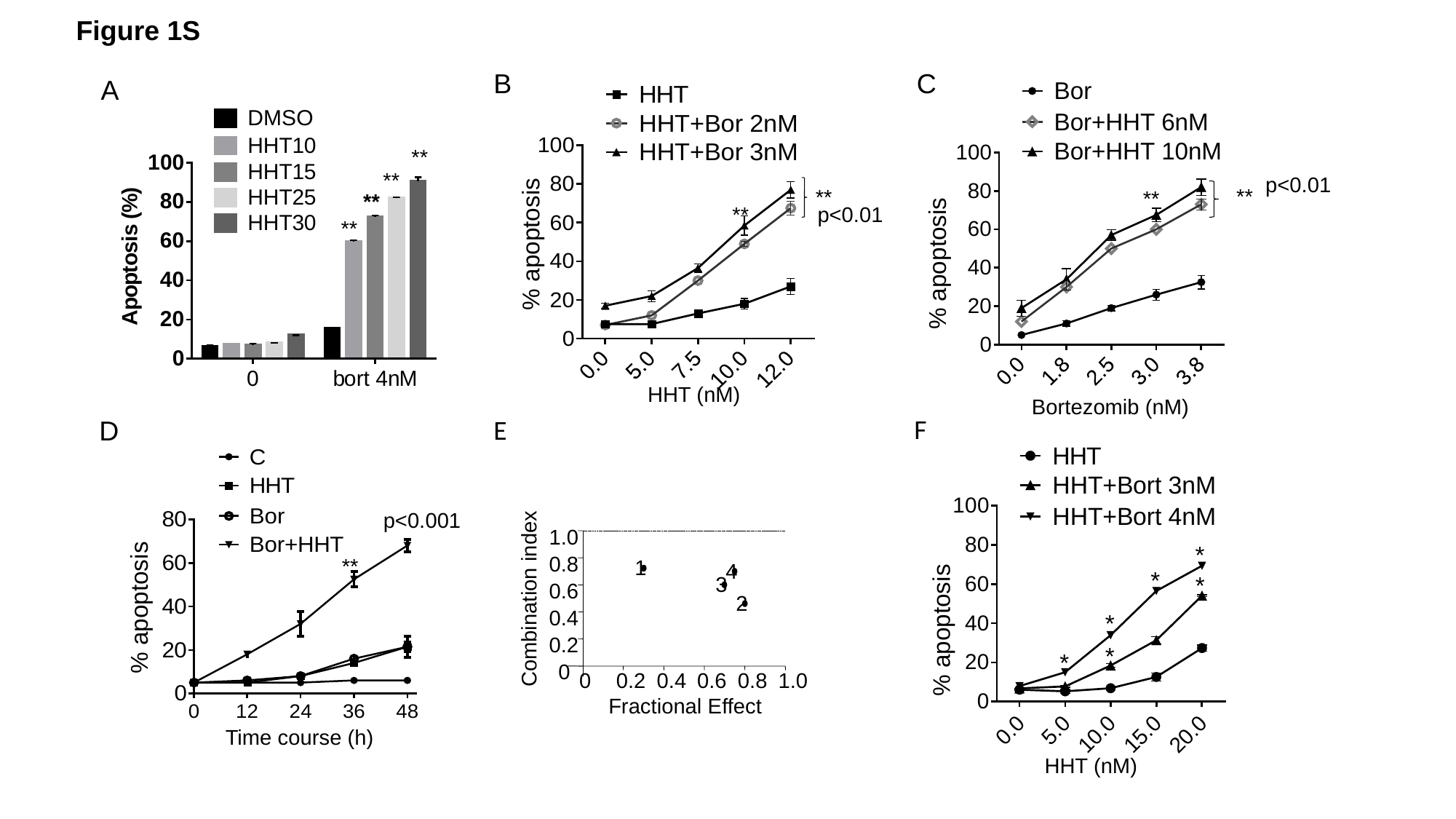

Figure 1S
C
B
A
**
**
p<0.01
% apoptosis
**
**
**
**
% apoptosis
**
p<0.01
**
HHT (nM)
Bortezomib (nM)
F
E
D
*
% apoptosis
*
*
*
*
*
HHT (nM)
1.0
0.8
1
4
3
0.6
Combination index
2
0.4
0.2
0
0
0.2
0.4
0.6
0.8
1.0
Fractional Effect
p<0.001
% apoptosis
**
Time course (h)
