# Supplementary material for: Homoharringtonine interacts synergistically with bortezomib in NHL cells through MCL-1 and NOXA-dependent mechanisms
Source: BMC Cancer. 2018 Nov 16;18:1129. doi: 10.1186/s12885-018-5018-x (PMC6240231; doi:10.1186/s12885-018-5018-x)
Supplement: Supplementary file 2 — The caspase inhibitor BOC-D-fmk does not change HHT/Bort–mediated down-regulation of MCL-1. OCI-LY18 and Carnaval cells were treated with HHT + Bort for 24 h either in the absence or presence of 5 μmol/L BOC-D-fmk. At the end of this period, cells were lysed and subjected to Western blot analysis using the indicated primary antibodies. Each lane was loaded with 25 μg of protein. Blots were stripped and reprobed with antitubulin antibodies to ensure equal loading and transfer of protein. Representative of two separate experiments. (PPTX 100 kb) [file 12885_2018_5018_MOESM2_ESM.pptx]

## Slide 1
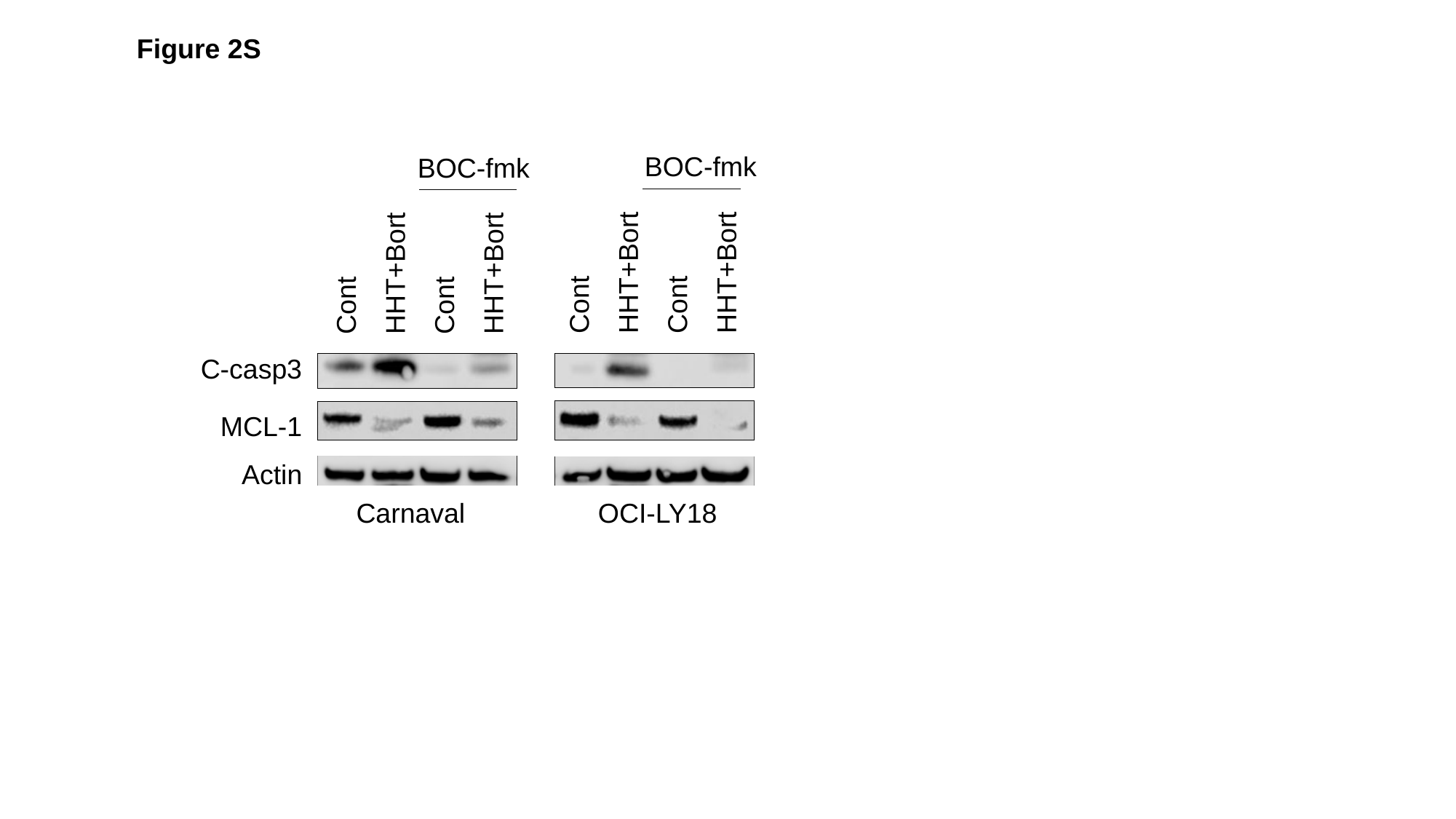

Figure 2S
BOC-fmk
BOC-fmk
Cont
HHT+Bort
Cont
HHT+Bort
Cont
HHT+Bort
Cont
HHT+Bort
C-casp3
MCL-1
Actin
Carnaval
OCI-LY18
