# Supplementary material for: Homoharringtonine interacts synergistically with bortezomib in NHL cells through MCL-1 and NOXA-dependent mechanisms
Source: BMC Cancer. 2018 Nov 16;18:1129. doi: 10.1186/s12885-018-5018-x (PMC6240231; doi:10.1186/s12885-018-5018-x)
Supplement: Supplementary file 3 — HHT inhibits MCL-1 expression through a post-transcriptional mechanism. A. SU-DHL4 and SU-DHL16 cells were treated with HHT for 8 h after which cells were lysed and proteins extracted. Expression of the indicated proteins was determined by Western blotting using the indicated antibodies. B. SU-DHL4 and SU-DHL16 cells were treated with HHT for 8 h after which cells were extracted for mRNA. Relative levels of MCL-1 mRNA/GAPDH were calculated. C. SU-DHL4 and SU-DHL16 cells were pre-treated with actinomycin (2.5 μg/ml) for 30 min and then exposed to HHT 2 h (SU-DHL4 60 nM, SU-DHL16 20 nM) after which cells were lysed and proteins extracted. Expression of the indicated proteins was determined by western blott using the indicated antibodies. D. SU-DHL4 and SU-DHL16 cells were pre-treated with cyclohexamide (5 μg/ml) for 30 min and then exposed to HHT 2 h and 4 h (SU-DHL4 60 nM, SU-DHL16 20 nM) after which cells were lysed and proteins extracted. Expression of the indicated proteins was determined by western blot. (PPTX 236 kb) [file 12885_2018_5018_MOESM3_ESM.pptx]

## Slide 1
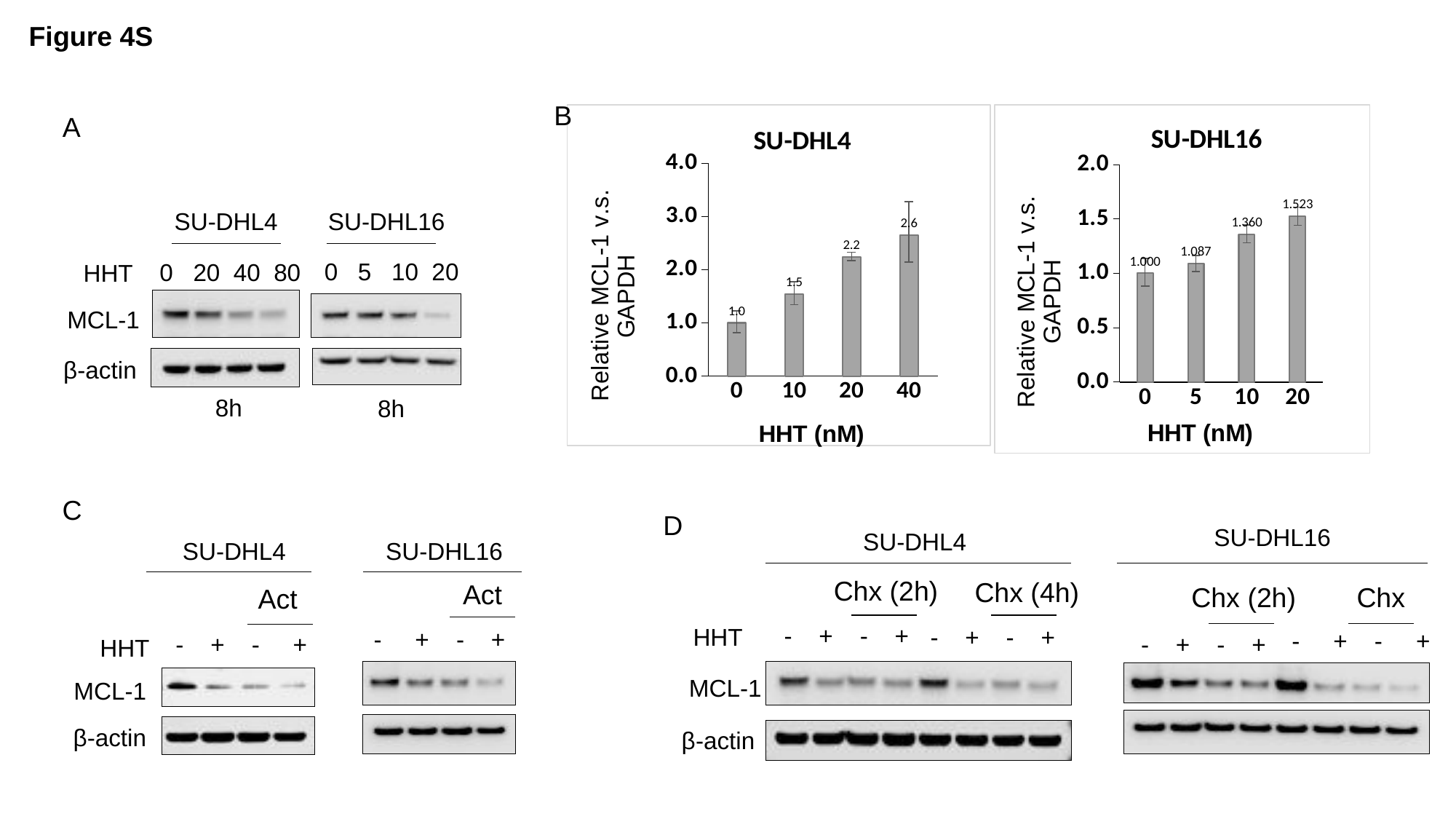

Figure 4S
B
### Chart: SU-DHL4
| Category | |
|---|---|
| 0 | 1.0 |
| 10 | 1.5423739472978912 |
| 20 | 2.2441886591099016 |
| 40 | 2.6465025914368 |
### Chart: SU-DHL16
| Category | |
|---|---|
| 0 | 1.0 |
| 5 | 1.0872959693379756 |
| 10 | 1.360200234863739 |
| 20 | 1.5231656302827936 |A
SU-DHL16
SU-DHL4
 0 5 10 20
0 20 40 80
HHT
MCL-1
β-actin
8h
8h
C
D
SU-DHL16
SU-DHL4
SU-DHL4
SU-DHL16
Chx (2h)
Chx (4h)
Act
- + - +
Chx (2h)
Chx
Act
- + - +
HHT
MCL-1
β-actin
- + - +
HHT
- + - +
- + - +
- + - +
MCL-1
β-actin
