# Supplementary figures and images for: Homoharringtonine interacts synergistically with bortezomib in NHL cells through MCL-1 and NOXA-dependent mechanisms
Source: BMC Cancer. 2018 Nov 16;18:1129. doi: 10.1186/s12885-018-5018-x (PMC6240231; doi:10.1186/s12885-018-5018-x)

## Slide 1
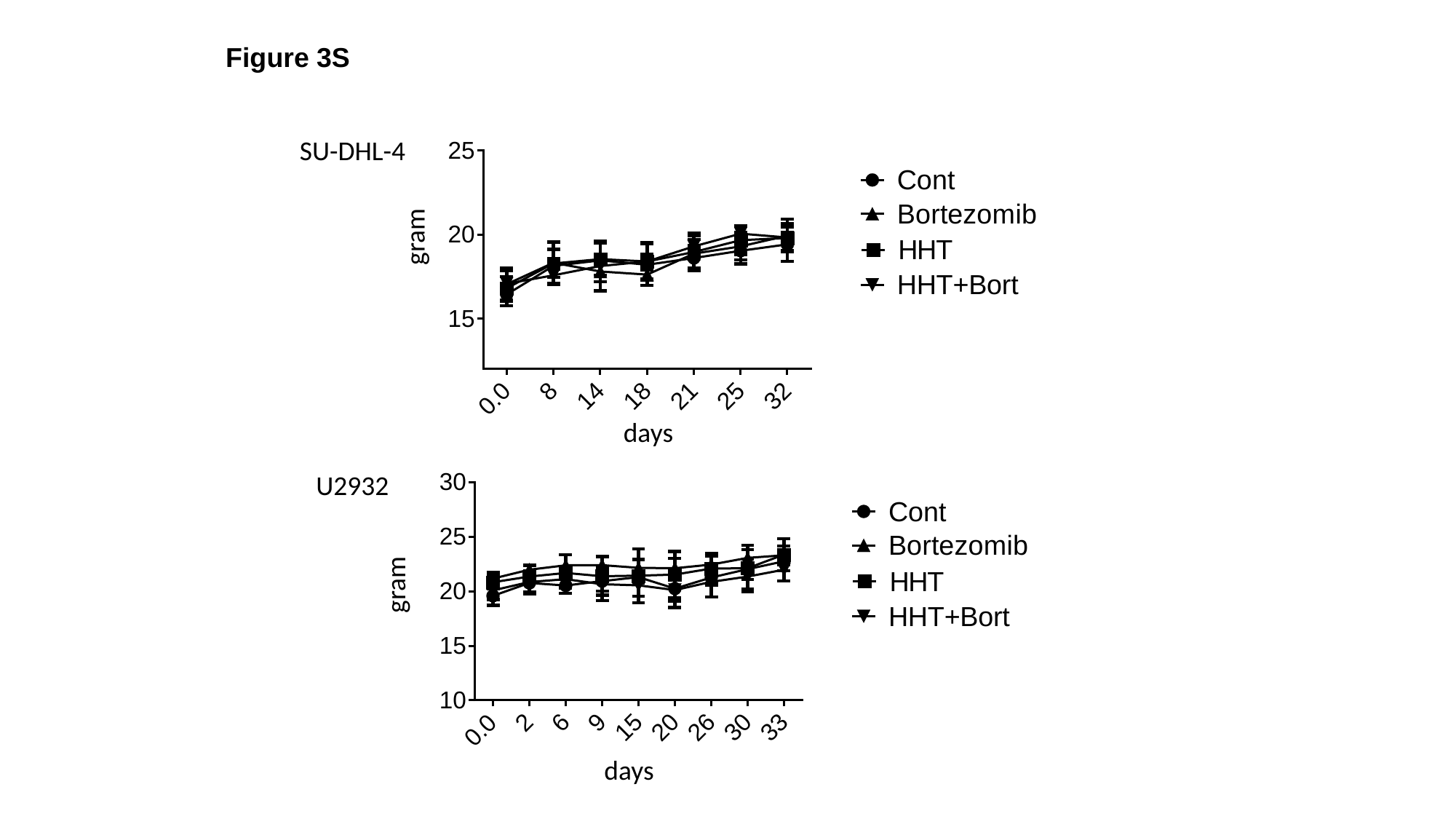

Figure 3S
SU-DHL-4
gram
days
U2932
gram
days

Supplement: Supplementary file 4 — Co-treatment with HHT and bortezomib does not cause significant weight loss in NSG mice. A. Weights of each mouse in the flank model study (SU-DHL-4) were monitored twice a week, and the mean weights for each group were plotted against days of treatment (p > 0.05 = no significant difference for combination group values compared to single-agent treatment or controls). B. Weights of each mouse in the systemic model study (U2932) were monitored twice a week and the mean weights for each group were plotted against days of treatment (p > 0.05 = no significant differences were noted for the combination group values compared to single-agent treatment or the control group. (PPTX 134 kb) [file 12885_2018_5018_MOESM4_ESM.pptx]
